# Comparison of efficacy of daily and alternate day maintenance treatment of GERD with Vonoprazan 10-mg using Gastroesophageal Reflux Disease Symptom Assessment Scale

**DOI:** 10.12669/pjms.40.4.8063

**Published:** 2024

**Authors:** Tazeen Rasheed, Bader Faiyaz Zuberi, Faiza Sadaqat Ali, Sana Muhammad Hussain, Pawan Kumar, Anoshia Saleem

**Affiliations:** 1 Tazeen Rasheed, FPCS. Associate Professor, Department of Medicine/Gastroenterology, Dow Medical College, Dow University of Health Sciences, Karachi, Pakistan; 2 Bader Faiyaz Zuberi, FCPS. Meritorious Professor, Consultant Gastroenterologist, OMI Hospital, Karachi, Pakistan; 3 Faiza Sadaqat Ali, FCPS. Senior Registrar, Department of Medicine/Gastroenterology, Dow Medical College, Dow University of Health Sciences, Karachi, Pakistan; 4 Sana Muhammad Hussain, FCPS. Fellow Gastroenterology, Department of Medicine/Gastroenterology, Dow Medical College, Dow University of Health Sciences, Karachi, Pakistan; 5 Pawan Kumar, FCPS. Assistant Professor, Department of Medicine/Gastroenterology, Dow Medical College, Dow University of Health Sciences, Karachi, Pakistan; 6 Anoshia Saleem, FCPS. Medical Officer, Department of Medicine, Dr Ruth KM Pfau Civil Hospital, Karachi, Pakistan

**Keywords:** Gastroesophageal Reflux Disease Symptom Assessment Scale (GSAS), GERD, Vonoprazan

## Abstract

**Objectives:**

To compare efficacy of 10-mg of vonoprazan daily & on alternate days by Gastroesophageal Reflux Disease Symptom Assessment Scale (GSAS)

**Method:**

This prospective interventional cohort was done at Department of Medicine/Gastroenterology Dow Medical College, Karachi, Pakistan during the period August 2022 & January 2023. Potential participants fulfilling inclusion and exclusion criteria were asked to fill out GSAS questionnaires after their written consent. Patients were allocated in to two groups using random tables. Group-A was given Tab Vonoprazan 10-mg daily for two weeks. Group-B was given Tab Vonoprazan 10-mg on alternate day. GSAS was scored by totaling scores across symptoms and then they are divided by the total number of non-missing symptom scores. Both groups were assessed week-0 & week-2.

**Results:**

Only 90 proformas that were completely filled were included, Group-A had 30 males and 15 females while Group-B had 29 males and 16 females. No significant difference in score was found in GSAS score at week-0 except that in item ‘gurgling’ while at week two there was no significant difference between any of the items. Total GSAS score were significantly lower at Week-2 than at week-0 (p = <.001).

**Conclusions:**

Vonoprazan of 10-mg on alternate day is equally effective as 10-mg daily in maintenance of GERD patients at two weeks.

## INTRODUCTION

Gastroesophageal reflux disease (GERD), is defined as a chronic condition that develops when the reflux of gastric contents into the proximal and distal esophagus causes symptoms and/or complications associated with it, affecting daily activity, and occurring at least twice a week.^1^ It occurs due to several mechanisms, which include lower esophageal sphincter incompetence, frequent relaxations of lower esophageal sphincter, hiatus hernia, impaired esophageal motility and obesity.^1^-^3^ All these mechanisms lead to excessive acid exposure to distal esophagus which may lead to mucosal damage.

This mucosal damage may be so subtle that it may be only visible on microscopy and histopathology also termed as microscopic esophagitis or endoscopically negative GERD. The inflammation may be gross and visible endoscopically as esophagitis which may lead to further progression to Barrett’s esophagitis. Barrett’s itself is a precursor to adenocarcinoma, thus GERD can have very serious consequences.^4^

The primary objective of a patient with GERD is to get relieve from its symptoms. To assess improvement or change in symptoms and quality of life of GERD patients is difficult and some scales are available for assessment. These include Health Related Quality of Life (HRQL). HRQL emphases on four major factors: physical and occupational function, psychological state, and social interaction. Most scales have used the physician as the input source. Physician data are acquired either by open-ended or semi-structured interview of the patient. Both methods are unreliable or result in misleading information. There is few GERD specific HRQL scales, but they suffer from narrowed approach towards GERD symptoms.

The Gastrointestinal Symptom Rating Scale (GSRS) was developed without patient input and thus is not illustrative of patient’s perspective of their condition. It does not integrate symptoms that are directly important to severity of disease like frequency of episodes, symptom severity and distress levels.^5^ A scale should incorporate information regarding severity, frequency and the most important component of distress. Gastroesophageal Reflux Disease Symptom Assessment Scale (GSAS) incorporates all these parameters.^5^ It was developed to determine treatment effects in the context of clinical trials. GSAS instrument has 15 items, limiting the scale to those symptoms with a minimum threshold of endorsement and relevance for the clinical trial Settings. It is the comprehensive evaluation scale of GERD symptoms.^6^ It is a 15-item tool that evaluates various aspects of GERD including stress about gastrointestinal symptoms.^7^

GSAS has been validated, it is stable and can detect changes in symptoms intensity that might occur over the period of time.^8^ Currently proton pump inhibitors (PPI) are treatment of choice for treatment of GERD but has to be taken on empty stomach and 30 minutes before meals.^9^,^10^ Recent introduction of potassium competitive acid blocker (P-CAB) have given better results due to the stronger, rapid and stable acid suppressing effect. They need not to be taken empty stomach and thus are more convenient to administer.^11^ To the best of our knowledge GSAS has not been used in Pakistan for assessment of GERD related symptoms with P-CAB on low dose of 10-mg daily neither on alternate days. In current study we intend to assess symptoms of GERD after 10-mg daily in group A and in group B, 10-mg on alternate days. This will help to determine the efficacy in low alternate dose and help in treatment cost of this disorder with patient and physician satisfaction.

### Operational definition

GERD will be labelled when reflux of gastric contents into the proximal and distal esophagus causes symptoms and/or complications associated with it, affecting daily activity, and occurring at least twice a week.^1^

### Study Instrument

Scoring of the GSAS scale is done on the presence of the symptoms and their bother ratings. Specifically, patients first indicate whether they had the symptom in the past week. If they did not have the symptom, then their score for the symptom is 0. If they did have the symptom, then they report how bothered they were by it on a 4-point scale (0=not at all, 1=somewhat, 2=quite a bit, 3=very much). The distress scale is scored by summing scores across symptoms and dividing by the total number of non-missing symptom scores. The GSAS is computed in this manner as long as 12 or more symptoms are scored. Patients with four or more missing symptom scores are assigned a missing GSAS distress score. The GSAS instrument is reproduced in Table-I.

**Table-I:** GSAS Instrument.

How much you were bothered by following symptoms of GERD during last week		Yes/No	0=Not at all	1=Somewhat	2=Quite a bit	3=Very much

#	Symptom					
	Heartburn					
	Pressure in Chest					
	Food back in moutd					
	Acid in mouth					
	Gurgling					
	Lump in throat					
	Nausea					
	Burning in throat					
	Bloating					
	Belching					
	Flatulence					
	Feeling full					
	Bad breath					
	Coughing					
	Hoarseness					

## METHOD

This prospective interventional cohort was done at Department of Medicine/Gastroenterology Dow Medical College, Karachi, Pakistan during the period August 2022 & January 2023. Potential participants who fulfilled inclusion and exclusion criteria were explained objectives and methods of study and were asked to respond to the GSAS questionnaires after their written consent. Patients were allocated in to two groups using random tables. Group-A was given Tab Vonoprazan 10-mg daily for two weeks. Group-B was given Tab Vonoprazan 10-mg on alternate day.

### Ethical approval

It was taken from IRB of Dow University of Health Sciences vide their letter # IRB-5289/DUHS/Approval/2022/923 dated 14-07-2022

Patients were required to indicate if they had symptoms in the last week, if they did not, then the score is reported as 0. If they did have the symptoms, then it was reported on the level of distress felt on a 3-point scale, where one is least and three is maximum. GSAS is scored by totaling scores across symptoms and then they are divided by the total number of non-missing symptom scores. Both groups were assessed week-0 & week-2 using GSAS.

Subjects were free to withdraw from the study at any time and with no consequences. Vonoprazan 10-mg (Vonoprazan^©^ Getz Pharmaceuticals (Pvt), Pakistan) is available in generic form by various pharmaceutical companies and the 10-mg dose is standard for maintenance of GERD and is available in public hospitals free of charge, in case if it is not available in local pharmacy, investigators provided it free by donation.

### Inclusion Criteria:

- Patients of both genders aged between 20-50 years complaining of symptoms of GERD.
- To be able to stay with the habitual customary diet.
- Having a BMI <30 kg/m^2^
- Non-smoker

### Exclusion criteria:

- Previous history of endoscopic therapy, GI Surgery or Barrett’s esophagus.
- Medical conditions as asthma, angina, liver, cardiovascular, immunological or kidney diseases.
- Taking drugs that might interfere in outcome of study or be a safety risk or perplex the interpretation of results, e.g., prokinetics, probiotics, and prebiotics.
- Pregnant or nursing women.
- Neoplasms.
- Patients with four or more missing symptoms of GSAS distress score were excluded.
- Patients using food supplements for gastrointestinal well-being.

### Sample Size Calculation

The sample size was calculated using PASS 2019 using *t-*test. The sample size was estimated considering the difference in GSAS scores. Data from the literature show that healthy participants have a GSAS score variability value of 12.5, using the value (*δ*) of clinically increase in score value by 10,^12^ an *α* value equal to 0.05, power of study was kept at 80%.^12^ These parameters resulted in a sample of 55 patients. A dropout rate of 8% was added to sample size, making the final sample size at 60 participants.

### Data Analysis

Demographic data like age & gender of selected patients was recorded. Mean ±SD of age and frequencies of gender were reported. Data of GSAS proforma were entered in SPSS at 0, 2 weeks. To determine whether GSAS reflect continuum of distress, the mean, standard deviation, and range of base line scores were evaluated. The internal consistency reliability was assessed using Cronbach’s alpha. This test is based on the average correlation among tested items in the scale. In the patients reporting minimal or no improvement in score on follow up, the test-retest reliability (reproducibility) was tested by computing intraclass correlation coefficients (ICC) between their baseline and follow-up GSAS scores using ANOVA.

Large values of ICCs specify greater agreement between repeated GSAS scores, where one represents complete agreement. Reliabilities >.70 were acceptable for group-level comparisons.^13^ Comparisons of GSAS scores at 0, two weeks were done with Paired Samples *t*-test in both groups, while comparisons of mean scores between Groups A & B at 0 & 2 weeks was done by Student’s *t*-test. Analysis was done using SPSS version 26. Significance level were set at ≤.05.

## RESULTS

One hundred eight patients returned the GSAS distress scale but only ninety forms that were filled completely were included. Forty-five patients were allocated to each group. Group-A had thirty males and fifteen females, while Group-B consisted of twenty-nine males and sixteen females. Mean age ±Standard Deviation (SD) according to gender and groups is given in Table-II. No significant difference in age among gender was detected by Student’s t-test [t (88) = -1.052; *p* =.296]. Internal validity of GSAS scale was assessed using Cronbach’s Alpha test was and found to be highly valid at .922.

**Table-II:** Mean age ±SD as per gender and groups.

	Group-A		Group-B	

	Male (n=30)	Female (n=15)	Male (n=29)	Female (n=16)
Age (yearsx̅ ±SD)	39.10 ±13.30	42.07 ±11.03	36.21 ±13.53	39.19 ±9.93

GSAS Scores were compared between two groups at Week-0 and Week-2, except for item ‘Gurgling’ no significant differences in scores were found at Week-0, at that time gurgling was significantly less in Group-A. At Week two there was no significant difference in any item between the two groups. Details are given in Table-III. Interval testing of scores within same group for difference in scores at week 0 & week-2 showed highly significant improvement of scores in both groups at p <.001. Table-III

**Table-III:** Mean GSAS Scores of between both groups with significance testing by Student’s t-test at induction (Week-0) and after two weeks of therapy (Week-two) and in same group for difference in scores at Week 0 & 2 by Paired Sample t-test.

Items	Groups	Week-0			Week-2			Difference (Week-0 - Week-2)	

		Mean	SD	Sig.	Mean	SD	Sig.	Diff.	Sig.
Heartburn	Group-A	2.18	0.81	.672	1.00	0.74	.400	1.18	<.001*
	Group-B	2.24	0.68		1.13	0.76		1.11	<.001*
Pressure in Chest	Group-A	1.67	0.98	.255	0.73	0.69	.440	0.94	<.001*
	Group-B	1.89	0.86		0.84	0.67		1.05	<.001*
Food back in mouth	Group-A	1.58	1.03	.185	0.60	0.72	.651	0.98	<.001*
	Group-B	1.84	0.85		0.67	0.67		1.17	<.001*
Acid in mouth	Group-A	1.96	0.90	.610	0.78	0.79	.890	1.18	<.001*
	Group-B	2.04	0.74		0.80	0.73		1.24	<.001*
Gurgling	Group-A	1.29	0.82	.040*	0.47	0.59	.148	0.82	<.001*
	Group-B	1.60	0.58		0.67	0.71		0.93	<.001*
Lump in throat	Group-A	1.33	0.98	.194	0.49	0.66	.999	0.84	<.001*
	Group-B	1.58	0.78		0.49	0.66		1.09	<.001*
Nausea	Group-A	1.51	0.87	.897	0.64	0.65	.870	0.87	<.001*
	Group-B	1.53	0.76		0.67	0.64		0.86	<.001*
Burning in Throat	Group-A	1.18	0.75	.387	0.64	0.57	.587	0.54	<.001*
	Group-B	1.31	0.70		0.71	0.59		0.6	<.001*
Bloating	Group-A	1.56	0.97	.631	0.67	0.77	.887	0.89	<.001*
	Group-B	1.64	0.77		0.64	0.71		1	<.001*
Belching	Group-A	1.36	0.77	.583	0.53	0.59	.853	0.83	<.001*
	Group-B	1.44	0.76		0.51	0.55		0.93	<.001*
Flatulence	Group-A	1.47	0.76	.683	0.76	0.80	.999	0.71	<.001*
	Group-B	1.53	0.79		0.76	0.68		0.77	<.001*
Feeling Full	Group-A	1.51	1.08	.595	0.73	0.91	.902	0.78	<.001*
	Group-B	1.62	0.89		0.71	0.79		0.91	<.001*
Bad Breath	Group-A	0.84	0.77	.661	0.40	0.50	.316	0.44	<.001*
	Group-B	0.91	0.67		0.51	0.55		0.4	<.001*
Coughing	Group-A	0.67	0.83	.284	0.29	0.46	.816	0.38	<.001*
	Group-B	0.84	0.74		0.27	0.45		0.57	<.001*
Hoarseness	Group-A	0.51	0.59	.261	0.07	0.25	.110	0.44	<.001*
	Group-B	0.64	0.53		0.18	0.39		0.46	<.001*
GSAS	Group-A	1.37	0.53	.163	0.59	0.41	.572	0.78	<.001*
	Group-B	1.51	0.41		0.64	0.34		0.87	<.001*

* . Significance level ≤.01.

## DISCUSSION

Our study showed that there was significant improvement in GSAS scores when assessed between Week-0 and Week-2 in both groups and no significant difference in GSAS Scores at Week-two between two groups showing that dose of Vonoprazan of 10-mg on alternate day is equally effective as 10-mg daily in maintenance of GERD patients. GERD is a chronic, recurrent disorder that requires appropriate, maintenance treatment to prevent recurrences. The main purpose of treating GERD is to bring the symptoms under control so that the individual feels better, heal the esophagus, prevent complications, and maintain the symptoms of GERD in remission so that daily life is unaffected by reflux and the patient does not develop refractory symptoms.^14^ It is fairly common in our part of the world and also in Middle Eastern region.^15^ The local guidelines have become obsolete due to introduction of new agents in management.^16^ Chronic reflux could lead many complications including erosive esophagitis and histopathological changes at lower esophageal junction.^17^

Vonoprazan has shown better efficacy in acid suppression in a cost-effective manner and has resulted in improvement in symptom resolution and have shown better results in helicobacter pylori eradication.^18^,^19^ Vonoprazan is studied against PPI too, in a study where comparison were made between alternate day vonoprazan and lansoprazole, vonoprazan was found to be superior in resolution of GERD symptoms.^11^ In another multicenter randomized crossover study from Japan vonoprazan on alternate day has shown to be equally effective and control of GERD and is cost-effective too.^20^ There has been concerns of hypergastrinemia with use of PPI and vonoprazan, as vonoprazan is more potent than PPI regarding acid suppression it is presumed to cause more severe hypergastrinemia.^21^ The alternate day regimen will alleviate such concerns and it gives similar results in terms of efficacy with cost savings.^20^

### Limitations

Study had limitation of being a single center study, more over long term safety of vonoprazan needs to be established by longer follow-up.

### Authors’ Contribution:

**TR:** Concept and design of study.

**BFZ:** Conceived study idea & gave final approval of the version to be published.

**FSA:** Data analysis and interpretations.

**SMH:** Data collection and responsible for data integrity.

**PK:** Critical revision of manuscript and responsible for data integrity.

**AS:** Drafting of manuscript.
